# Morphology-engineered alleviation of mycelial aggregation in *Streptomyces* chassis for potentiated production of secondary metabolites

**DOI:** 10.1016/j.synbio.2025.05.010

**Published:** 2025-05-26

**Authors:** Shuo Liu, Fei Xiao, Lanxin Lv, Meiyan Wang, Wenli Li, Guoqing Niu

**Affiliations:** a College of Agronomy and Biotechnology, Southwest University, Chongqing, 400715, China; b Institute of Biotechnology, Shanxi University, Taiyuan, 030006, Shanxi, China; c Key Laboratory of Marine Drugs, Ministry of Education, School of Medicine and Pharmacy, Ocean University of China, Qingdao, 266003, China; d State Key Laboratory for Crop Stress Resistance and High-Efficiency Production, Shaanxi Key Laboratory of Natural Products & Chemical Biology, College of Chemistry & Pharmacy, Northwest A&F University, Yangling, Shannxi, 712100, China

**Keywords:** Morphology engineering, *Streptomyces coelicolor*, Actinorhodin, Staurosporine, Carotenoid

## Abstract

The genus *Streptomyces* exhibits a complex life cycle of morphological differentiation and an extraordinary capacity to produce numerous bioactive secondary metabolites. In submerged cultures, *Streptomyces* species usually grow in the form of mycelial networks and aggregate into large pellets or clumps, which is generally unfavorable for industrial production. This study aimed to construct efficient microbial cell factories by manipulating morphology-related genes. We herein employed a morphology engineering approach to generate eight engineered derivatives (MECS01∼MECS08) of *Streptomyces coelicolor* M1146, a versatile chassis widely used for the heterologous production of various secondary metabolites. We found that genetic manipulation of morphology-related genes exerted a substantial influence on the growth and mycelial characteristics of the engineered strains. Once the native actinorhodin gene cluster was introduced into these strains, antibiotic production increased in all engineered strains compared to the parental strain. Notably, a significant elevation of actinorhodin production was observed in three of the engineered strains, MECS01, MECS03 and MECS05. Similar scenarios occurred when expressing the staurosporine gene cluster and the carotenoid gene cluster in these three engineered derivatives, respectively. Our study demonstrates that morphology engineering represents an effective strategy for alleviating mycelial aggregation. It has also expanded the toolkit of *Streptomyces* chassis available for the heterologous expression of gene clusters encoding a variety of secondary metabolites.

## 1 Introduction

The genus *Streptomyces* consists of filamentous bacteria that undergo a complex multicellular life cycle and propagate *via* sporulation. This cycle progresses from vegetative growth to the formation of reproductive aerial hyphae, which then differentiate into long chains of spores [[1], [2], [3]]. This characteristic is strikingly similar to that of filamentous fungi. The life cycle of *Streptomyces* species features two clearly distinguishable filamentous cell forms: the growing (or vegetative) hyphae and the reproductive (or aerial) hyphae. It is not surprising that *Streptomyces* species form extensive branching substrate and aerial mycelia [4]. The development of *Streptomyces* on surface-grown cultures has been extensively investigated. The cellular morphogenesis of *Streptomyces* is governed by a complex network of genes, which precisely coordinates and controls various morphological changes of these bacteria. Over the past few decades, substantial efforts have been dedicated to dissect the *bld* genes that initiate aerial growth and the *whi* genes that govern spore formation [1,[4], [5], [6]]. It is noteworthy that multiple studies have identified genetic elements related to mycelial architecture. For example, *matAB* and *cslA/glxA* encode proteins involved in the biosynthesis of extracellular polymeric substances (EPSs). In model organisms such as *Streptomyces coelicolor* and *Streptomyces lividans*, the MatAB proteins catalyze the formation of extracellular poly-β-1,6-*N*-acetylglucosamine (PNAG) [7,8]. The CslA and GlxA proteins are thought to be responsible for synthesizing cellulase-like EPS polymers, featuring β-(1–4) glycosidic bonds at hyphal tips and branch sites [9,10]. However, the exact structure of this polysaccharide remains elusive. Moreover, SsgA has been recognized as an essential determinant in sporulation, as it induces hyphae fragmentation by promoting septum formation [11]. During sporulation, *Streptomyces* species undergo a large-scale cell division process. In this phase, the formation of sporulation septa ladders transforms multigenomic hyphae into chains of unigenomic spores. This process depends on cytokinetic Z-rings assembled by FtsZ, the bacterial tubulin homolog [12,13].

In recent years, the development of engineered hosts, particularly those with minimized genomes from which a specific set of biosynthetic gene cluster (BGCs) has been removed, has attracted increasing interest [14,15]. Model organisms, such as *S. coelicolor*, *S*. *lividans*, *Streptomyces avermitilis*, and *Streptomyces albidoflavus*, have been the focus of such engineering endeavors. The first engineered host, *S. coelicolor* CH999, was generated through the deletion of the *act* gene cluster and the introduction of mutations in the *red* gene cluster, rendering it deficient in the production of actinorhodin (ACT) and prodiginine (RED) [16]. More sophisticated surrogate hosts have been derived from the model strain *S. coelicolor* [17,18]*.* For instance, *S. coelicolor* M1146 was constructed by eliminating the biosynthetic gene clusters for ACT, RED, CDA, and CPK from *S. coelicolor* M145. Further introduction of point mutations in *rpoB* and *rpsL* resulted in the creation of *S. coelicolor* M1152 and *S. coelicolor* M1154, respectively. These strains have been successfully employed for high-level production of ACT, chloramphenicol, congocidine, and novobiocin [18,19]. It is worth noting that engineering efforts have extended beyond model organisms. For instance, *Streptomyces chattanoogensis* L320 and L321, engineered derivatives of the industrial natamycin-producing strain *S. chattanoogensis* L10 [20], exemplify such efforts. Moreover, an engineered polyketide-focused chassis strain derived from *Streptomyces* sp. A4420 [21] serves as additional example, further highlighting the progress achieved in the engineering of non-model organisms. These well-characterized hosts, each boasting distinct features, serve as versatile chassis for the expression of BGCs dedicated to the generation of novel natural products.

For the production of bioactive specialized metabolites, submerged batch fermentation represents a commonly-utilized and highly effective technique for cultivating *Streptomyces* species. However, the intricate morphology of *Streptomyces* poses significant challenges on its ability to maximize product yields. Unlike fermentations with unicellular microorganisms, such as *Saccharomyces cerevisiae*, *Escherichia coli*, and *Bacillus subtilis*, *Streptomyces* species tend to form aggregated mycelial pellets or clumps when grown in submerged cultures [22]. These morphological characteristics not only impede mass transfer processes but also affect nutrient uptake, thereby complicating efforts to achieve high-yield production of valuable metabolites. This situation presents a major challenge for industrial applications, as the large-scale production of secondary metabolites predominantly occurs in bioreactors [22]. A similar phenomenon was observed in filamentous fungi, where engineered hyphal dispersion significantly enhanced secondary metabolite production [23,24]. Notably, while the development of *Streptomyces* on surface-grown cultures has been thoroughly explored, the factors regulating morphogenesis in submerged cultures remain poorly understood. This complexity in morphology highlights the need for tailored strategies to overcome these constraints and boost the efficiency of *Streptomyces*-based fermentations. Unfortunately, despite the significance of this issue, only a single investigation has been conducted to address the problem of mycelial aggregation in *Streptomyces*. This study demonstrated that deletion of the *matAB* locus in *S. coelicolor* M1152 effectively mitigated mycelial aggregation. This genetic modification was accompanied by improved biomass accumulation and enhanced anthracyclinone production [25].

Currently, there is still an ever-increasing interest in the development of versatile chassis for the heterologous expression of a variety of BGCs. In this study, we engineered eight derivatives of *S. coelicolor* M1146 through targeted manipulation of cell morphology-related genes. Our morphology engineering strategy centered on deleting genes like *matAB* and *cslA/glxA*, as well as overexpressing genes such as *ssgA* and *ftsZ*, either individually or in combination. Compared to *S. coelicolor* M1146, all engineered strains MECS01∼MECS08 exhibited a substantial reduction in mycelial aggregation. When the actinorhodin gene cluster (*act*) was introduced, these eight engineered strains demonstrated enhanced antibiotic production compared to *S. coelicolor* M1146. Notably, three of the engineered strains, MECS01, MECS03 and MECS05, showed a significant increase in actinorhodin production. Similar results were obtained when the staurosporine gene cluster (*spc*) was heterologously expressed in these three engineered strains. Furthermore, when an engineered gene cluster for carotenoid biosynthesis (*crt*) was expressed in these three engineered derivatives, a significant increase in titer was also observed. These engineered chassis show great promise for the heterologous expression of BGCs encoding a broad spectrum of secondary metabolites, thereby providing novel tools for natural product discovery and engineered production.

## 2 Materials and methods

### 2.1 Bacterial strains and culture conditions

A complete list of bacterial strains is provided in Table S1 of the Supplementary Material. *Escherichia coli* DH5α was used as a general host for routine subcloning procedures. *E. coli* ET12567 (pUZ8002) was employed to transfer DNA from *E. coli* to *Streptomyces* through intergeneric conjugation. *E. coli* BW25113 (pIJ790) was used for constructing recombinant plasmids *via* λ-Red-mediated recombination technology [[26], [27]]. The cultivation and manipulation of *E. coli* strains were performed following standard protocols [28]. *Streptomyces* strains were typically cultivated on mannitol soya flour (MS) agar or in yeast extract-malt extract (YEME) liquid medium [29]. *S*. *coelicolor* strains were routinely grown at 28 °C either on agar plates or in liquid cultures. Liquid cultures were performed in siliconized flasks with shaking at 220 rpm.

### 2.2 Plasmids construction

Plasmids and primers are listed in Tables S2 and S3 of the Supplementary Material, respectively. For the construction of pKCcas9::Δ*matAB*, the upstream and downstream fragments flanking the *matAB* locus were PCR-amplified from *S. coelicolor* M1146 genomic DNA by using primer pairs matAB UpF/UpR and matAB DnF/DnR, respectively. A fragment containing *sgRNA* was obtained from pKCcas9dO with the primer pair matAB sgRNA F/R. The *sgRNA* fragment was assembled with the upstream fragment by using overlap extension PCR with primers matAB sgRNA F and matAB UpR, and the assembled fragment was cut with *Spe*I/*Afl*II. The downstream fragment was digested with *AflII*/*Hin*dIII. These two fragments were ligated together into *Spe*I/*Hin*dIII double-digested pKCcas9dO to obtain pKCcas9::Δ*matAB*. A similar strategy was used for the construction of pKCcas9::Δ*cslA*/*glxA*. To generate pKCcas9::Δ*matAB*-*ssgA*OE, a fragment containing *ssgA* driven by the *hrdB* promoter was inserted between the upstream and downstream fragments of pKCcas9::Δ*matAB via* homologous recombination technology. A similar strategy was employed for the construction of pKCcas9::Δ*cslA*/*glxA*-*ftsZ*OE.

### 2.3 Strain construction and confirmation

Once verified through restriction digestion, the recombinant plasmids were first introduced into *E. coli* ET12567/pUZ8002. Subsequently, they were transferred into *S. coelicolor* M1146 through *E. coli*-*Streptomyces* intergeneric conjugation [29]. The mutant strains were obtained *via* CRISPR/Cas9-mediated genome editing [30]. Genomic DNAs were then extracted from candidate mutants and then served as templates for verification by PCR amplifications with specific primers in Table S3 of the Supplementary Material.

### 2.4 Light microscopy

*S. coelicolor* M1146 and its engineered derivatives were inoculated into three different liquid media, including the commonly used YEME, YEME with sucrose omitted or TSB liquid media. Samples were taken at different time points, and were mounted onto glass slides, and then dried under a heating lamp. The samples were fixed with formaldehyde for 30 s. After being washed with ddH_2_O, the glass slides were covered with crystal violet solution for 1 min. The crystal violet solution was then washed off with ddH_2_O. Once again, the slides were dried under the heating lamp, and the dried slides were observed under a Nikon ECLIPSE Ei light microscope. For measurement of mycelial length and pellet diameter, *S. coelicolor* M1146 and its engineered derivatives were cultivated in YEME for 3 days. Following crystal violet staining, 100 hyphae were randomly selected per visual field for length measurement using ImageJ software. Concurrently, diameters of all mycelial pellets within the same field were measured using the same software.

*Streptomyces* spores were collected with water and then filtered through cotton wool. Subsequently, the optical density of the spore suspension was measured at a wavelength of 450 nm (OD_450_), and each suspension was adjusted to an OD_450_ of 1.0. A 10 μL aliquot of the normalized spore suspension was spotted on MM, AS-1, and R2YE agar plates. These plates were cultivated at 28 °C for 5 days. *Streptomyces* colonies on the plates were observed using a Zeiss SteREO Discovery V20 stereomicroscope.

### 2.5 Scanning electron microscopy

*S*. *coelicolor* M1146 and its derivatives were cultivated on MM or R2YE media at 28 °C for 5 days. The agar plates were sliced into samples measuring 6 mm × 6 mm. These samples were then immersed in glutaraldehyde at 4 °C for 8 h. Subsequently, the glutaraldehyde fixative was aspirated, and the samples were rinsed with deionized water successively. Specifically, the samples were soaked in deionized water for 6 min, followed by a second soaking in fresh deionized water for 7 min, and then a third soaking in deionized water for 8 min. Next, the deionized water was removed, and the samples were immersed in 50 % ethanol for 14 min. Subsequently, 50 % ethanol was aspirated, and the samples were soaked in 70 % ethanol for another 14 min. This process was repeated with 85 % ethanol and 95 % ethanol, each for 14 min. Finally, the samples were immersed in anhydrous ethanol for 15 min, and this step was replicated three times to ensure complete dehydration. Once the sample preparation was finished, observations were carried out using a Hitachi cold field emission scanning electron microscope SU8010.

### 2.6 Measurement of cell growth

The biomass of *S*. *coelicolor* M1146 and its derivatives was determined following the protocol as described previously [31]. Briefly, spore suspension of each strain was inoculated into 10 mL liquid YEME and cultivated for 48 h to obtain a seed culture. Subsequently, 1.0 mL of the seed culture was transferred into 50 mL of YEME. Samples (0.5 mL each) were collected by centrifugation at 12,000 *g* for 5 min. The pellets were washed with 0.5 mL sterilized water and then dissolved in the diphenylamine reaction buffer. The mixture was vortexed for 1 min and then kept at 60 °C for 1 h, followed by centrifugation at 12,000 *g* for 1 min. OD_595_ of the supernatants were measured with a multimode Varioskan LUX microplate reader (Thermo Scientific).

### 2.7 Engineering of the staurosporine biosynthetic gene cluster

To construct pBS::*hrdB*-*spc*UpDn, the upstream fragment of the *spc* BGC was amplified by PCR from pWLI617 [32] by using primer pair spc UpF/R. The neomycin phosphotransferase gene (*neo*) was amplified with primer pair spc neo F/R from pUC119::*neo*. Subsequently, the upstream fragment was assembled with the *neo* cassette by using overlap extension PCR with primers spc UpF and spc neo R. The resulting amplicon was cut with *Xba*I/*Eco*RI, and inserted into the corresponding sites of pBluescript II KS (+) to obtain pBS::*spc*Up-*neo*. Next, the *hrdB* promoter was amplified from pSET152::*hrdB*-*neo* with primer pair spc hrdB pF/R [33]. The downstream fragment of the *spc* BGC was amplified from pWLI617 [32] by using primer pair spc DnF/R. The *hrdB* promoter was cut with *Eco*RI/*Nde*I, and the downstream fragment was cut with *Nde*I/*Xho*I. The two fragments were ligated together into *Eco*RI/*Xho*I double-digested pBS::*spc*Up-*neo* to generate pBS::*hrdB*-*spc*UpDn. To engineer the gene cluster, the fragment containing upstream and downstream regions, the *neo* cassette and the *hrdB* promoter was released from pBS::*hrdB*-*spc*UpDn with *Xba*I/*Xho*I, and used to replace the original promoter of *spcR* with the *hrdB* promoter *via* λ-Red-mediated recombination technology [26].

### 2.8 Cloning and engineering of the carotenoid biosynthetic gene cluster

We employed a strategy based on multiplex amplification-and-ligation to obtain the carotenoid biosynthetic gene cluster (*crt* BGC). In brief, three fragments were amplified by using genomic DNA from *S. coelicolor* M1146 as template with primer pairs crt 1F/1R, crt 2F/2R, and crt 3F/3R, respectively. The PCR amplicons were digested with appropriate restriction enzymes and then assembled by iterations of restriction and ligation to generate pBS::*crt*. The upstream and downstream fragments flanking the *crt* gene cluster were amplified from *S. coelicolor* M1146 genomic DNA by using primer pairs crt UpF/UpR and crt DnF/DnR, respectively. Next, the *hrdB* promoter was amplified from pSET152::*hrdB*-*neo* with the primer pair hrdB UpF/hrdB crt pR [33]. The *hrdB* promoter was then assembled with the upstream fragment by using overlap extension PCR with primers hrdB UpF and crt UpR, and the resulting amplicon was cut with *Xba*I/*Bgl*II. In the meantime, the *hrdB* promoter was amplified from pSET152::*hrdB*-*neo* with the primer pair hrdB DnF/hrdB crt pR [33]. Next, the *hrdB* promoter was assembled together with the downstream fragment by using overlap extension PCR with primers with hrdB DnF and crt DnR, and the resulting amplicon was cut with *BglII*/*Eco*RI. The two fragments were ligated together into *Xba*I/*Eco*RI double-digested pSET152 to generate pSET152::*hrdB-crt*UpDn. The *crt* gene cluster was transferred into pSET152 *via* λ-Red-mediated recombination with *Bgl*II linearized pSET152::*hrdB-crt*UpDn to obtain pSET152::*hrdB-crt* [26].

### 2.9 Measurement of actinorhodin production

Quantification of actinorhodin production was performed as described previously [34]. In brief, *S. coelicolor* M1146 and the engineered strains were cultivated in 50 mL of R5MS. Subsequently, 1 mL culture was collected at different time intervals and treated with KOH (1 N final concentration). Actinorhodin quantification was then carried out by measuring the absorbance at 640 nm [35].

### 2.10 Production and analysis of staurosporine

Production and analysis of staurosporine was performed as described previously with minor modifications [32]. In brief, spores of *Streptomyces* strains were inoculated in 50 mL of TSBY medium and incubated for 48 h to obtain the seed culture. Next, 5 mL of the seed culture was transferred into 50 mL of fermentation medium (1.5 % soybean meal, 0.5 % yeast extract, 0.2 % soluble starch, 0.2 % peptone, 0.4 % NaCl and 0.4 % CaCO_3_, pH 7.3) and cultivated for 5 days. The supernatant and the cell pellets were separately collected through centrifugation. Subsequently, 40 mL acetone was added to the cell pellets, and the mixture was sonicated for 40 min to facilitate the extraction. The acetone phase was then collected by centrifugation and then evaporated using a rotary evaporator. The remaining aqueous phase was combined with the supernatant of the fermentation broth. The combined solution was extracted with twice its volume of ethyl acetate. The ethyl acetate phase was collected, evaporated using a rotary evaporator, and then the resulting residue was dissolved in 1.5 mL methanol. The methanol suspension was filtered through a Millipore membrane with a pore diameter of 0.22 μm. High-performance liquid chromatography (HPLC) analysis was performed with an Agilent 1260 Infinity HPLC system equipped with a 1260 DAD VL detector and a YMC-Pack ODS column (5 μm, 12 nm, 150 × 4.6 mm). The mobile phase consists of ACN + 0.1 % trifluoroacetic acid (TFA) and ddH_2_O + 0.1 % trifluoroacetic acid (TFA), and the flow rate was set at 1 mL/min. The elution program was as follows: 0–5 min, 20 % ACN + 0.1 % TFA; 5–25 min, 20–60 % ACN + 0.1 % TFA; 26–35 min, 100 % ACN + 0.1 % TFA. The detection wavelength is 294 nm. Quantification of staurosporine production was achieved by using a curve generated from samples of authentic standard.

### 2.11 Production and analysis of carotenoids

Production and analysis of carotenoids was performed as described previously with minor modifications [36]. For carotenoids production, spore suspensions were inoculated in 10 mL of YEME liquid media and incubated for 48 h as seed culture. One milliliter of seed culture was then transferred into 50 mL of M79 liquid media. The fermentation broths were withdrawn at different time intervals and collected by centrifugation at 13,000 *g* for 10 min. The pellets were resuspended in methanol containing 6 % KOH and incubated at 60 °C for 15 min. The mixtures were then extracted with equal volume of petroleum ether and absorbance at 370 nm–500 nm was recorded.

## 3 Results

### 3.1 Construction of chassis strains utilizing the versatile *S*. *coelicolor M1146*

**Fig. 1:**
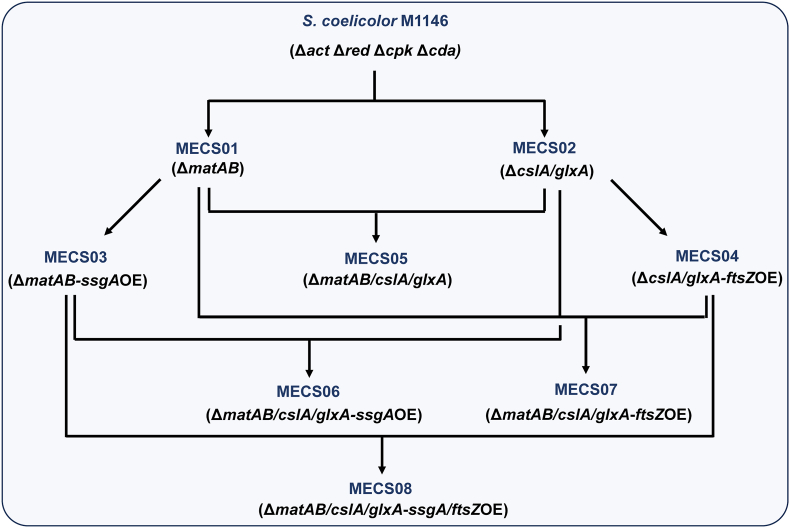
A simplified schematic illustration for the construction of the engineered chassis strains. All eight strains were generated *via* CRISPR/Cas9-mediated genome engineering of *S. coelicolor* M1146. These strains were designated as Morphology Engineering Chassis Strains (MECS01∼08). The genes targeted for deletion were *matAB* and *cslA*/*glxA*, and the genes selected for overexpression were *ssgA* and *ftsZ*.

As mentioned earlier, three engineered strains, *S*. *coelicolor* M1146, M1152 and M1154, have been extensively used for the heterologous production of diverse secondary metabolites [37]. In a previous study, deletion of the *matAB* locus from *S. coelicolor* M1152 resulted in the generation of an enhanced cell factory, *S. coelicolor* M1152Δ*matAB*. This engineered strain has been used to improve the production of anthracyclinone [25]. To construct more efficient microbial cell factories, our attention was directed to the genetic manipulation of additional morphology-related genes. In addition to *matAB*, *cslA/glxA* was chosen based on the fact that CslA and GlxA proteins assemble into a functional cellulose synthase complex that is responsible for cellulose biosynthesis at growing tips [38]. Similar to *matAB*, *cslA/glxA* is also implicated in the aggregation process of liquid-grown *Streptomyces* cultures [10]. We anticipate that the deletion of the two gene loci will disrupt extracellular polymers, thereby reducing mycelial aggregation. To this end, we employed CRISPR/Cas9-mediated genome editing to knock-out *matAB* and *cslA/glxA* in *S*. *coelicolor* M1146, leading to the creation of MECS01 (Δ*matAB*) and MECS02 (Δ*cslA*/*glxA*) (Fig. 1 and S1). These strains were designated as Morphology Engineering Chassis Strains (MECS). Moreover, SsgA plays a crucial role in stimulating mycelial fragmentation through the promotion of septum formation [11,39]. Additionally, FtsZ serves as a key determinant in the formation of both vegetative cross-walls and sporulation septa [40]. The overexpression of *ssgA* and *ftsZ* has the potential to induce hyphal compartmentalization, thereby mitigating mycelial aggregation. Further modifications generated six additional mutant strains (Fig. 1). For example, MECS03 (Δ*matAB*-*ssgA*OE) was generated by introducing the overexpression of *ssgA* in the MECS01 background. Similarly, MECS04 (Δ*cslA*/*glxA*-*ftsZ*OE) was obtained through the overexpression of *ftsZ* in MECS02 (Fig. 1 and S1). Through a combination of gene deletion and overexpression strategies, four strains, such as MECS05 (Δ*matAB*/*cslA*/*glxA*), MECS06 (Δ*matAB*/*cslA*/*glxA*-*ssgA*OE), MECS07 (Δ*matAB*/*cslA*/*glxA*-*ftsZ*OE), and MECS08 (Δ*matAB*/*cslA*/*glxA*-*ssgA*/*ftsZ*OE), were constructed. Thus, a total of eight engineered strains were obtained with *S*. *coelicolor* M1146 as the parental strain (Fig. 1). All engineered strains were verified by PCR amplifications (Fig. S2).

### 3.2 Comparison of mycelial morphology between *S*. *coelicolor* M1146 and the engineered derivatives in submerged cultures

**Fig. 2:**
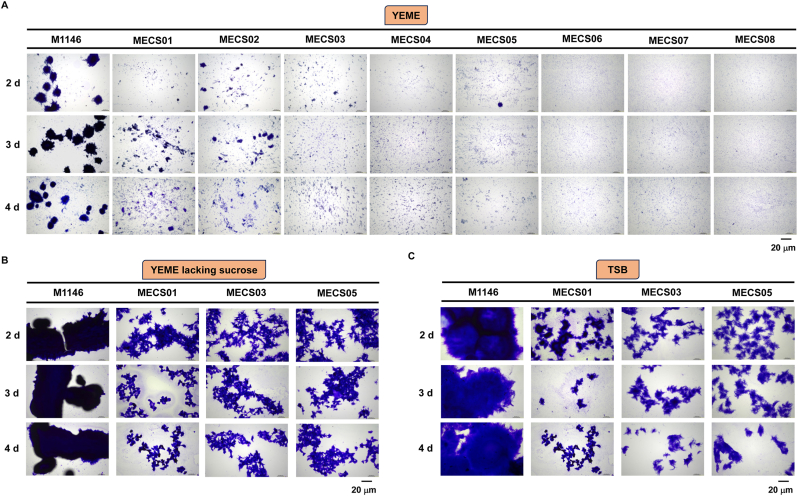
Microscopic images of *S. coelicolor* M1146 and its engineered derivatives grown in sub-merged conditions. (A) *S. coelicolor* M1146 (M1146) and eight engineered strains (MECS01∼08) cultured in liquid YEME medium for 2, 3, and 4 days. (B) *S. coelicolor* M1146 (M1146), MECS01, MECS03, and MECS05 strains grown in liquid YEME omitting sucrose medium for 2, 3, and 4 days. (C) *S. coelicolor* M1146 (M1146), MECS01, MECS03, and MECS05 strains grown in TSB medium for 2, 3, and 4 days. Scale bar: 20 μm.

Next, mycelial morphology of *S*. *coelicolor* M1146 and the engineered strains were examined under submerged cultures conditions in three different cultivation media. When cultivated in liquid YEME media, which contains 34 % sucrose typically used to disperse *Streptomyces* culture, significant morphological differences were observed among these strains over a 3-day period, spanning from Day 2 to Day 4. Microscopic observations showed that *S*. *coelicolor* M1146 (hereafter abbreviated as M1146) formed large and aggregated mycelial clumps even in the presence of 34 % sucrose. In striking contrast, MECS01 and MECS02 produced mycelial clumps with markedly smaller particle sizes (Fig. 2A). Quantitative analysis revealed that the mean mycelial length of MECS01 (5.87 ± 2.95 μm) was longer than that of M1146 (4.33 ± 1.82 μm), whereas the mycelial length of MECS02 (1.03 ± 0.37 μm) exhibited a significant decrease in mycelial length compared to M1146 (Fig. S3A). Moreover, quantitative analysis demonstrated that the average pellet diameters of MECS01 (9.67 ± 3.51 μm) and MECS02 (1.21 ± 0.52 μm) was significantly smaller than that of M1146 (29.02 ± 6.34 μm) (Fig. S3B). MECS03, which lacks both *matAB* and *cslA*/*glxA*, demonstrated a remarkable decrease in mycelial length and particle size (Fig. 2A and Fig. S3). Similar observations were made with the other engineered strains, particularly MECS06-MECS08 which exhibited sparser and finer mycelial distributions (Fig. 2A and Fig. S3).

Furthermore, MECS01, MECS03 and MECS05 were selected for cultivation either in liquid YEME medium omitting sucrose or TSB liquid medium. Compared to those cultivated in YEME, both M1146 and the engineered strains formed conspicuously larger mycelial clumps. However, all engineered strains displayed a significant reduction in particle size relative to M1146 (Fig. 2B and C). In the sucrose-free YEME medium, M1146 retained its relatively compact mycelial aggregates, while MECS01, MECS03, and MECS05 exhibited more dispersed and branched mycelial morphologies (Fig. 2B). When cultivated in TSB medium, M1146 formed dense and large-sized mycelial clumps, whereas MECS01, MECS03, and MECS05 presented fragmented and smaller mycelial clusters (Fig. 2C). These results clearly indicate that different culture media have a substantial impact on the mycelial morphology of these strains. This suggests that morphology engineering represents an effective strategy for controlling mycelial aggregation during the submerged cultivation of *Streptomyces*. To assess the impact of genetic manipulations on cellular growth, the growth curves of M1146 and the engineered strains were monitored over a period from 48 h to 120 h. The results showed that MECS06 demonstrated the most robust growth among all the strains, followed by MECS07 and MECS05. In contrast, M1146 exhibited relatively sluggish growth pattern (Fig. S4).

### 3.3 Morphological characteristics of *S*. *coelicolor* M1146 and the engineered derivatives on agar plates

**Fig. 3:**
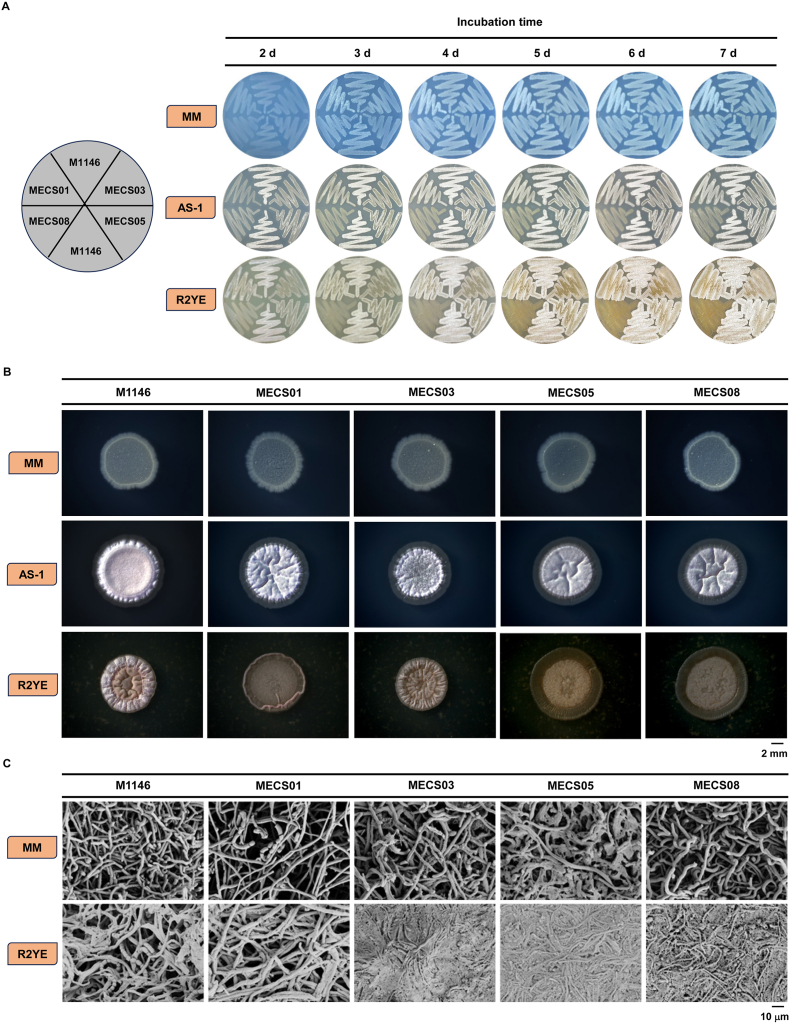
Growth and morphological characteristics of *S. coelicolor* M1146 and the engineered strains cultivated on different agar media. (A) Images of *S. coelicolor* M1146, MECS01, MECS03, MECS05, and MECS08 strains cultured on MM, AS-1 and R2YE media for 2–7 days. (B) Macroscopic views of colonies of different strains on MM, AS-1, and R2YE media for 5 days. Scale bar: 2 mm. (C) Scanning electron microscopy images showing the morphological details of strains on MM and R2YE media. Scale bar: 10 μm.

To assess the morphological traits, M1146 and four engineered strains (MECS01, MECS03, MECS05, and MECS08) were selected for cultivation on three commonly used agar media, MM, AS-1 and R2YE. After 3 days of growth on MM agar plates, the confluent lawn of M1146 turns grey. In contrast, MECS01 formed a white lawn within the same incubation period, and this white color persisted even after a prolonged incubation of 7 days (Fig. 3A). However, the lawns of the remaining engineered strains resembled that of M1146. When cultivated on AS-1, all engineered strains displayed morphological appearances distinct from M1146 with a particularly significant difference observed in MECS08. Similar observations were made when the strains were cultivated on R2YE plates (Fig. 3A). Stereo microscopy analysis showed no significant difference between M1146 and its engineered derivatives when cultivated on MM plates. On AS-1 plates, the colony of M1146 presented a fuzzy and smooth middle ring, while the engineered strains displayed more intricate surface patterns. Compared to M1146, the surfaces of the engineered strains, especially MECS01, MECS05, and MECS08, were wrinkled (Fig. 3B). A distinctive colony morphology was also evident when the strains were cultivated on R2YE plates (Fig. 3B). Additionally, scanning electron microscopy revealed that mycelial structures of M1146 were more robust and evenly branched, while those of the engineered strains differed in density and branching patterns. On R2YE plates, the mycelia of MECS03, MECS05 and MECS08 appeared sparser and less interconnected than M1146 (Fig. 3C). Overall, these results clearly demonstrated that genetic manipulation of morphology-related genes exert a substantial impact on the growth and mycelial characteristics of the engineered strains.

### 3.4 Screening the chassis strains for boosted actinorhodin production

**Fig. 4:**
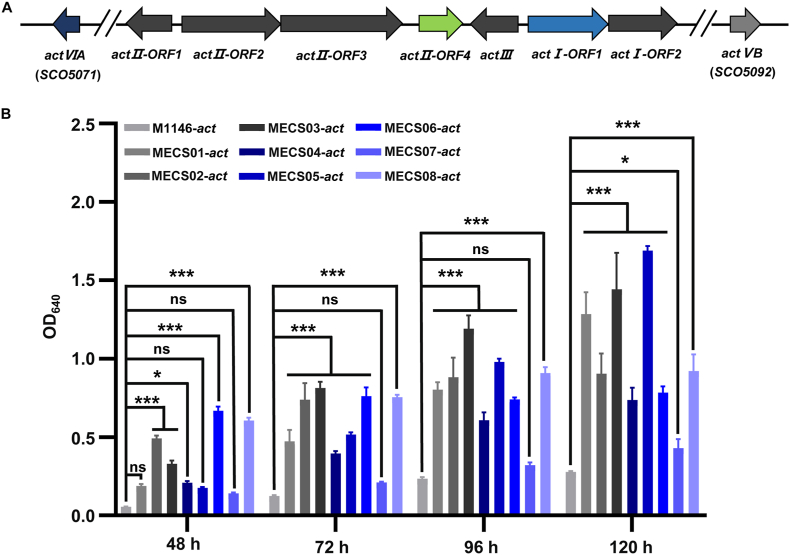
Actinorhodin production in *S. coelicolor* M1146 and the engineered strains. (A) Genetic organization of the *act* BGC in *S. coelicolor*. The arrows represent different open-reading frames (ORFs) within the gene cluster, and the corresponding gene names and locus tags (in parentheses for *actVIA* and *actVB*) are indicated. (B) Comparison of actinorhodin production levels in *S. coelicolor* M1146 and the eight chassis strains. The y-axis shows the optical density at 640 nm (OD_640_), which reflects the amount of actinorhodin produced. The x-axis represents different time points of 48 h, 72 h, 96 h, and 120 h. Error bars indicate standard deviations. “ns” denotes no significant difference, while “∗” and “∗∗∗” represent significant differences at different levels according to statistical analysis (*P* < 0.05 and *P* < 0.001 respectively).

To compare the engineered strains in terms of secondary metabolites production, we started with the well-characterized actinorhodin biosynthetic gene cluster (*act* BGC) from *S. coelicolor* M145. The gene cluster encodes an archetypal type II polyketide actinorhodin (ACT), covering a continuous region of 25 kb from SCO5071 to SCO5092 (Fig. 4A). In a previous study, the entire *act* BGC was captured into pSET152 to generate pSET152::*act* [33]. The recombinant plasmid pSET152::*act* was then introduced into the eight engineered derivatives of M1146. The resulting strains were grown in R5MS for ACT quantification. The results revealed that there are significant differences in ACT production among strains at different time intervals (Fig. 4B). For example, at 72 h and 96 h, except for MECS06, the engineered strains show highly significant differences when compared to M1146-*act*. As time progresses to 120 h, all engineered strains exhibit significant differences in their ACT production. Notably, at an incubation time of 120 h, the highest ACT production was detected in MECS05-*act*, followed by MECS03-*act*, and MECS01-*act*. These results suggested that introduction of the *act* BGC led to enhanced antibiotic production in the engineered strains, especially in the mutant strain lacking both *matAB* and *cslA/glxA* loci. Based upon these findings, MECS01, MECS03, and MECS05 were selected for subsequent investigations.

### 3.5 Chassis strains as hosts for heterologous production of staurosporine

**Fig. 5:**
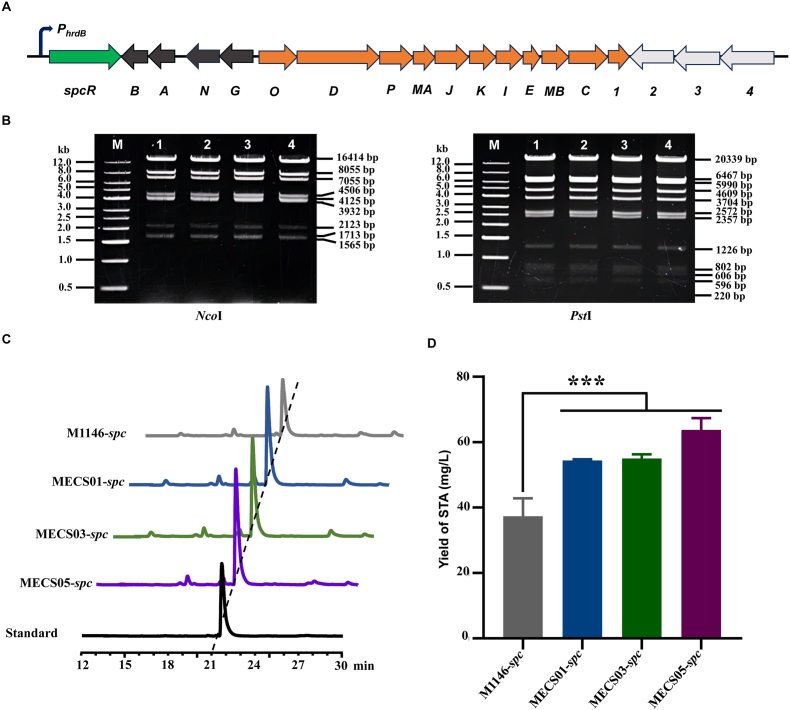
Production of staurosporine in *S. coelicolor* M1146 and its engineered derivatives. (A) Genetic organization of the *spc* BGC in *S*. *sanyensis* FMA. The native promoter of *spcR* was replaced with the *hrdB* promoter. The arrow indicates the transcription direction. (B) Restriction digestion analysis of the engineered gene cluster with *Nco*I (left) and *Pst*I (right). M represents the DNA marker, and 1–4 represent different samples of the engineered gene cluster. The sizes of DNA fragments (in base pairs) are indicated on the right side of each image. (C) Chromatograms comparing the samples from M1146-*spc*, MECS01-*spc*, MECS03-*spc*, and a standard sample. The x-axis represents time in min, showing the separation profile of components. (D) Yield of staurosporine in fermentation broth of different strains. Error bars represent standard deviations, and “∗∗” indicates a significant difference at *P* < 0.01. STA: staurosporine.

To assess the production efficiency of the engineered strains regarding other secondary metabolites, an engineered staurosporine gene cluster (*spc*) was introduced into M1146, along with MECS01, MECS03, and MECS05. The *spc* BGC governs the biosynthesis of staurosporine, which is a member of the indolocarbazole alkaloid family. In prior studies, a 32 kb DNA region harboring this gene cluster was cloned from marine-derived *Streptomyces sanyensis* FMA and successfully expressed in surrogate host *S*. *coelicolor* M1146 [32,41]. Of note is that a similar gene cluster was also identified in *Streptomyces fradiae* CGMCC 4.576 [42]. StaR, a cluster-situated LAL family regulator, promotes staurosporine production by binding to the promoter regions of *staO*-*staC* and *staG*-*staN* in *S. fradiae* CGMCC 4.576 [42]. We speculate that SpcR, a homolog of StaR, functions as an activator of the *spc* BGC in a similar manner. To facilitate staurosporine production, the native promoter of *spcR* was replaced with the constitutive *hrdB* promoter *via* λ-Red-mediated recombination (Fig. 5A and S5). The resulting recombinant plasmid pSET152AB::*hrdB*-*spc* was confirmed by restriction digestion (Fig. 5B), and introduced into M1146, MECS01, MECS03 and MECS05 to obtain M1146-*spc*, MECS01-*spc*, MECS03-*spc*, and MECS05-*spc*, respectively. To compare the productivity of staurosporine, all strains were cultivated in fermentation medium for staurosporine quantification. The results revealed that all engineered strains produced more staurosporine than the parental strain (Fig. 5C). Specifically, MECS01-*spc* and MECS03-*spc* exhibited comparable yields, with each achieving approximately 1.5-fold increases relative to M1146-*spc*. Notably, MECS05-*spc* demonstrated the highest production level, showing an approximate 1.7-fold increase compared to M1146-*spc* (Fig. 5D). These results suggested that the introduction of the *spc* gene cluster led to enhanced staurosporine production within the three engineered strains. Of note is that MECS05 exhibited the most optimal performance in staurosporine production, which is consistent with our previous findings regarding ACT.

### 3.6 Comparative analysis of the chassis strains for carotenoid production

**Fig. 6:**
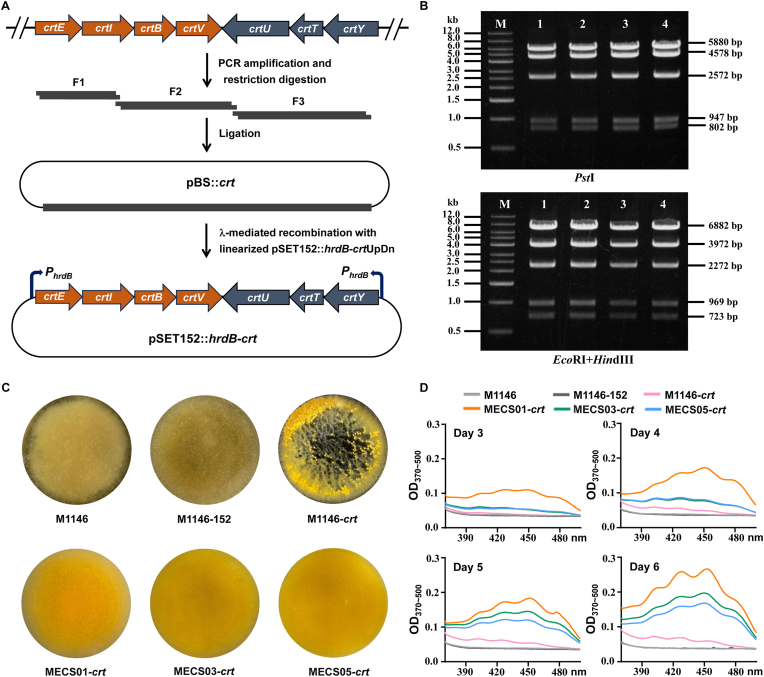
Production of carotenoid in *S. coelicolor* M1146 and its engineered derivatives. (A) Schematic illustration for the cloning and engineering of the *crt* BGC. Three fragments (F1, F2 and F3) were PCR-amplified and assembled in a defined order into pBluescript II KS(+) to obtain pBS*crt*. The *crt* BGC was then transferred into pSET152 with *Bgl*II linearized pSET152*hrdB*-*crt*UpDn to generate pSET152*hrdB*-*crt*. (B) Gel electrophoresis results of restriction digestion either with *Pst*I (the upper gel) or with *Eco*RI and *Hin*dIII (the lower gel). M represents the DNA marker, and 1–4 represent different samples of the engineered gene cluster with indicated fragment sizes in base pairs. (C) Heterologous expression of the *crt* BGC in *S. coelicolor* M1146 and the engineered strains. All strains were cultivated in shaking flasks containing M79 for 6 days. The bright yellow color was only observed with culture broths of recombinant strains containing the *crt* BGC. The image was taken from the upside of the flasks. (D) Absorbance spectra at OD_370_–OD_500_ showing production of carotenoid in different strains measured at time intervals as indicated.

To further expand the diversity of the secondary metabolites, we opted to investigate compounds belonging to the terpenes family. In a previous study, a carotenoid biosynthetic gene cluster (*crt* BGC) was identified in the genome of *S. coelicolor* A3(2). The *crt* BGC consists of two convergent transcriptional unites, *crtEIBV* and *crtYTU*. Expression of the gene cluster is controlled by the promoters preceding *crtE* and *crtY*, which in turn depends on a MerR family transcriptional regulator LitR and an extracytoplasmic function (ECF) sigma factor LitS [43]. To this end, we employed a strategy based on multiplex amplification-and-ligation to clone the *crt* BGC. Three fragments were PCR-amplified and assembled together by iterations of restriction and ligation to generate pBS::*crt* (Fig. 6A). In a similar manner, the promoters of *crtE* and *crtY* were substituted with the constitutive *hrdB* promoter *via* λ-Red-mediated recombination to facilitate carotenoid production (Fig. 6A). The resulting recombinant plasmid pSET152::*hrdB*-*crt* was verified by restriction analysis (Fig. 6B), and then introduced into M1146, MECS01, MECS03, and MECS05 to obtain M1146-*crt*, MECS01-*crt*, MECS03-*crt*, and MECS05-*crt*, respectively. M1146 and M1146-pSET152 were used to serve as negative controls, where M1146-pSET152 is a derivative of M1146 harboring the empty plasmid pSET152. All strains were cultivated in M79 medium for carotenoid comparison. The production of carotenoid was first evaluated through the visualization of the characteristic yellow color in the fermentation broths (Fig. 6C). Quantitative analysis showed that MECS01-*crt*, MECS03-*crt*, and MECS05-*crt* produced more carotenoid than M1146-*crt* (Fig. 6D), suggesting that introduction of the engineered *crt* BGC led to enhanced carotenoid production in the three engineered strains. These results further validated that manipulating mycelial aggregation is an effective approach for enhancing carotenoid production.

## 4 Discussion

Filamentous actinobacteria of the genus *Streptomyces* possesses two remarkable features: a complex life cycle featuring morphological differentiation and an outstanding capacity to produce diverse bioactive metabolites. During submerged fermentation, *Streptomyces* species typically grow as mycelial networks and form large pellets. These pellets may impede the diffusion of nutrients and the excretion of metabolites, ultimately reducing the production efficiency of target products [8]. To address this issue, a previous study demonstrated that deleting the *matAB* locus from *S. coelicolor* M1152 resulted in an enhanced cell factory, which was successfully used to boost the production of anthracyclinone [25]. In a recent study, deletion *matAB* in *S. coelicolor* A3(2) led to a significant increase in its conjugation efficiency [44]. Here, our study focused on constructing more efficient microbial cell factories by manipulating *matAB* and several other genes associated with cell morphology. We found that the deletion of the *matAB* and *cslA*/*glxA* genes, along with overexpression of the *ssgA* and *ftsZ* genes, significantly mitigated mycelial aggregation in the engineered strains. When cultivated in different liquid media, the engineered strains exhibited smaller mycelial pellet sizes and more dispersed distributions in comparison to M1146 (Fig. 2). This clearly indicates that the morphology engineering strategy can effectively regulate mycelial aggregation during the liquid cultivation of *Streptomyces*.

We speculate that the observed alterations in mycelial aggregation are mainly attributed to disruptions in *matAB* and *cslA*/*glxA*. As mentioned earlier, *matAB* and *cslA*/*glxA* encode proteins responsible for the biosynthesis of PNAG and cellulase-like EPS polymers, respectively. Extracellular accumulation of PNAG enables *Streptomyces* to adhere to hydrophilic glass surfaces, while attachment to hydrophobic polystyrene surfaces is mediated by the cellulase-like EPS polymers [8]. Notably, PNAG-dependent adhesion to hydrophilic surfaces is kinetically rapid, with measurable attachment evident in overnight cultures. In contrast, hydrophobic surface adhesion *via* cellulase-like EPS polymers is a slow process, requiring week-long incubations to detect significant attachment. Disruption of *matAB* impairs the production of PNAG, a major component of extracellular matrix that mediates hyphal adhesion to hydrophilic glass surface [8]. These results highlight the functional specialization of MatAB- and CslA-dependent EPS in mediating substrate- and time-scale-specific adhesion strategies. However, further studies are required to provide more direct evidence of the correlation between these EPSs and mycelial aggregation.

A thorough analysis of the growth and mycelial characteristics of the engineered strains on different agar plates indicated that genetic manipulation exerted a significant impact on the growth and morphology of these strains. In contrast to M1146, the engineered strains displayed distinct colony morphologies and mycelial structures (Fig. 3), further highlighting the pivotal role of morphology-related genes in regulating the growth and development of *Streptomyces*. These differences might be related to factors such as the strains’ nutrient utilization efficiency and environmental adaptability, both of which are worthy of further investigation. Furthermore, it would be of great interest to investigate other well-characterized genes associated with cell morphology.

From the perspective of secondary metabolite production, the introduction of the native actinorhodin gene cluster, the engineered staurosporine gene cluster, and the engineered carotenoid gene cluster led to significant increases in the production of corresponding metabolites in the engineered strains. Notably, the enhancement of metabolite production was more prominent in three engineered strains, MECS01, MECS03, and MECS05 (Fig. 4, Fig. 5, Fig. 6). This suggests that the *Streptomyces* chassis cells optimized by morphology engineering can provide a more favorable cellular environment for the synthesis of secondary metabolites, promoting the efficient operation of metabolic pathways. We speculate that dispersed mycelial morphology may enhance nutrient accessibility and uptake efficiency by increasing the hyphae surface area. In the meanwhile, this morphology facilitates more efficient metabolite export, thereby making the intracellular metabolic balance more conducive to the synthesis of target products. However, the specific molecular mechanisms by which morphology-related genes regulate secondary metabolite synthesis remain poorly understood. It is also critical to acknowledge that we cannot exclude the possibility that mycelial pellets might promote the production of certain secondary metabolites. Nutrient-limiting microenvironments within pellets could potentially impose stress on cells, thereby activating secondary metabolic pathways. Exploring this hypothesis across diverse classes of secondary metabolites would be a compelling avenue for future investigations. Moreover, our study was mainly focused on chassis strain derived from the model organism. It would be interesting to assess the application potential of morphology engineering in non-model strains, especially those industrial strains. However, expanding this approach to non-model systems requires addressing two main challenges. The lack of efficient transformation systems remains a primary obstacle. Many industrial *Streptomyces* strains exhibit low competence for exogenous DNA, impeding targeted editing of morphology-related genes. Overcoming this necessitates the development of strain-specific genetic platforms, such as novel plasmid delivery methods (protoplast transformation) or optimized CRISPR-Cas9 systems [45,46]. Another challenge is the scale-up morphological variability. Industrial bioreactors impose shear stresses (e.g., high impeller speeds, turbulent flow) that may have profound impact on morphological structures of the engineered strains. Pilot-scale studies under simulated industrial conditions are critical to validate the scalability of morphology-engineered strains in industrial bioproduction. Despite these challenges, these efforts will lay a more robust foundation for the extensive application of *Streptomyces* chassis in fields such as biopharmaceuticals and agriculture.

## 5 Conclusion

In this study, we successfully engineered eight derivatives of *S*. *coelicolor* M1146 through morphology engineering. These engineered strains demonstrated significant differences in mycelial morphology compared to the parental strain. Moreover, upon the introduction of three distinct secondary metabolite gene clusters, the production of actinorhodin, staurosporine, and carotenoids was increased. This clearly indicates that manipulating cell morphology-related genes represents an effective strategy for alleviating mycelial aggregation and optimizing *Streptomyces* chassis cells for secondary metabolite production. However, further in-depth research and exploration are needed to clarify the molecular mechanisms underlying the impact of morphology engineering on secondary metabolite biosynthesis. Moreover, it is of great importance to evaluate the application potential of morphology engineering in non-model industrial strains for large-scale industrial production of bioactive secondary metabolites.

## CRediT authorship contribution statement

**Shuo Liu:** Writing – review & editing, Validation, Methodology, Investigation. **Fei Xiao:** Writing – review & editing, Validation, Methodology, Investigation. **Lanxin Lv:** Validation, Investigation. **Meiyan Wang:** Validation, Investigation. **Wenli Li:** Writing – review & editing, Supervision, Funding acquisition, Conceptualization. **Guoqing Niu:** Writing – review & editing, Writing – original draft, Supervision, Funding acquisition, Conceptualization.

## Funding

This study was supported in part by a grant from the 10.13039/501100012166National Key Research and Development Program of China (No. 2023YFD1700700), and grants from the 10.13039/501100001809National Natural Science Foundation of China (No. 32370077, U22A20582, 32100051), the Shaanxi Provincial Science and Technology Projects (2024RSCXTD-67 and QCYRCXM-2023-040).

## Declaration of competing interest

The authors declare that they have no known competing financial interests or personal relationships that could have appeared to influence the work reported in this paper.
