# Corrigendum to “Morphology-engineered alleviation of mycelial aggregation in *Streptomyces* chassis for potentiated production of secondary metabolites” [Synth Syst Biotechnol 10 (3) (2025) 1059–1069]

**DOI:** 10.1016/j.synbio.2025.11.006

**Published:** 2025-12-18

**Authors:** Shuo Liu, Fei Xiao, Lanxin Lv, Meiyan Wang, Wenli Li, Guoqing Niu

**Affiliations:** a College of Agronomy and Biotechnology, Southwest University, Chongqing, 400715, China; b Institute of Biotechnology, Shanxi University, Taiyuan, 030006, Shanxi, China; c Key Laboratory of Marine Drugs, Ministry of Education, School of Medicine and Pharmacy, Ocean University of China, Qingdao, 266003, China; d State Key Laboratory for Crop Stress Resistance and High-Efficiency Production, Shaanxi Key Laboratory of Natural Products & Chemical Biology, College of Chemistry & Pharmacy, Northwest A&F University, Yangling, Shaanxi, 712100, China

The authors regret that following the publication of the original article, the authors noticed that some of the images in Fig. 2 were incorrect. Below is the corrected figure.

**Fig. 2:**
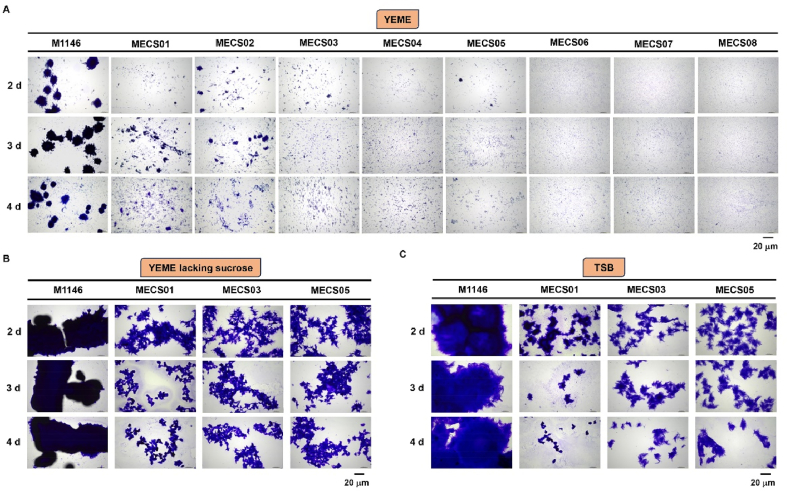
Microscopic images of *S. coelicolor* M1146 and its engineered derivatives grown in sub-merged conditions. (A) *S. coelicolor* M1146 (M1146) and eight engineered strains (MECS01∼08) cultured in liquid YEME medium for 2, 3, and 4 days. (B) *S. coelicolor* M1146 (M1146), MECS01, MECS03, and MECS05 strains grown in liquid YEME omitting sucrose medium for 2, 3, and 4 days. (C) *S. coelicolor* M1146 (M1146), MECS01, MECS03, and MECS05 strains grown in TSB medium for 2, 3, and 4 days. Scale bar: 20 μm.

The authors would like to apologize for any inconvenience caused.

