# Supplementary material for: Morphology-engineered alleviation of mycelial aggregation in Streptomyces chassis for potentiated production of secondary metabolites
Source: Synth Syst Biotechnol. 2025 May 26;10(3):1059–69. doi: 10.1016/j.synbio.2025.05.010 (PMC12173525; doi:10.1016/j.synbio.2025.05.010)
Supplement: Multimedia component 1 [file mmc1.docx]

Supplementary Material

**Morphology-engineered alleviation of mycelial aggregation in *Streptomyces* chassis for potentiated production of secondary metabolites**

Shuo Liu^1,#^, Fei Xiao^3,#^, Lanxin Lv^1^, Meiyan Wang^1^, Wenli Li^3, 4,*^, and Guoqing Niu^1,2,*^

^1^ College of Agronomy and Biotechnology, Southwest University, Chongqing, 400715, China

^2^ Institute of Biotechnology, Shanxi University, Taiyuan, 030006, Shanxi, China.

^3^ Key Laboratory of Marine Drugs, Ministry of Education, School of Medicine and Pharmacy, Ocean University of China, Qingdao 266003, China

^4^ State Key Laboratory for Crop Stress Resistance and High-Efficiency Production, Shaanxi Key Laboratory of Natural Products & Chemical Biology, College of Chemistry & Pharmacy, Northwest A&F University, Yangling, Shannxi 712100, China

^#^ These authors contributed equally to this work.

***Corresponding authors**

Guoqing Niu

E-mail address: niu062376@swu.edu.cn

orcid.org/0000-0002-4796-5630

Wenli Li

E-mail address: liwenli@nwafu.edu.cn

orcid.org/0000-0003-1598-3217

# Supplementary Figures

**
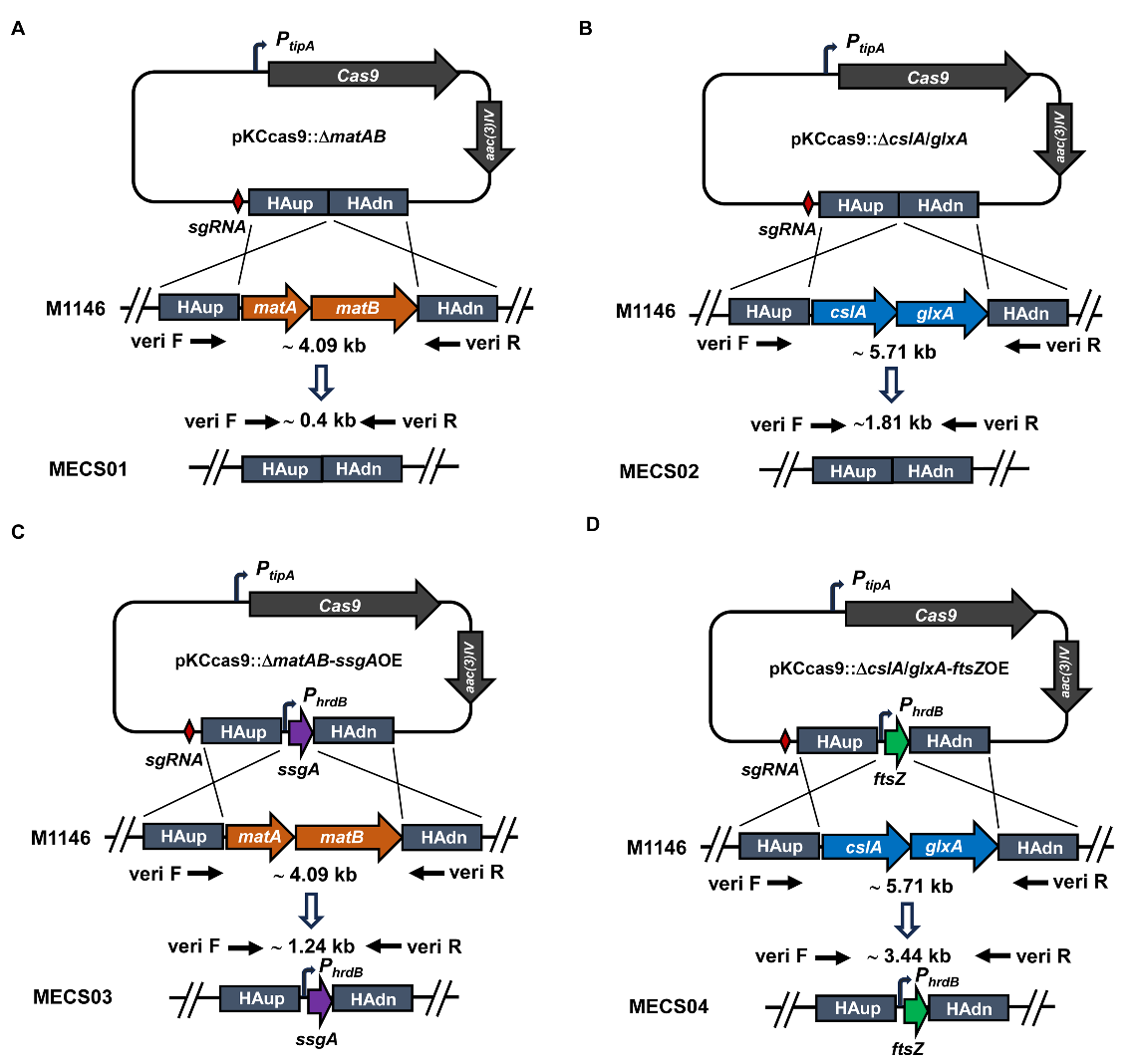
**

**Figure S1. Schematic illustrations of CRISPR/Cas9-mediated genome engineering for the construction of MECS01~04.** (A) Schematic representation of CRISPR/Cas9-mediated knock-out of *matAB*. (B) Schematic representation of CRISPR/Cas9-mediated knock-out of *cslA*/*glxA*. (C) Schematic representation of CRISPR/Cas9-mediated knock-out of *matAB* with simultaneous knock-in of *ssgA* under the control of the *hrdB* promoter. (D) Schematic representation of CRISPR/Cas9-mediated knock-out of *cslA*/*glxA* with simultaneous knock-in of *ftsZ* under the control of the *hrdB* promoter. *S. coelicolor* M1146 (M1146) and the four engineered strains were subjected to DNA extractions and subsequent PCR amplifications. The expected sizes of PCR amplicons are as indicated.“kb” represents kilobase; *P_hrdB_* represents the constitutive *hrdB* promoter.

**
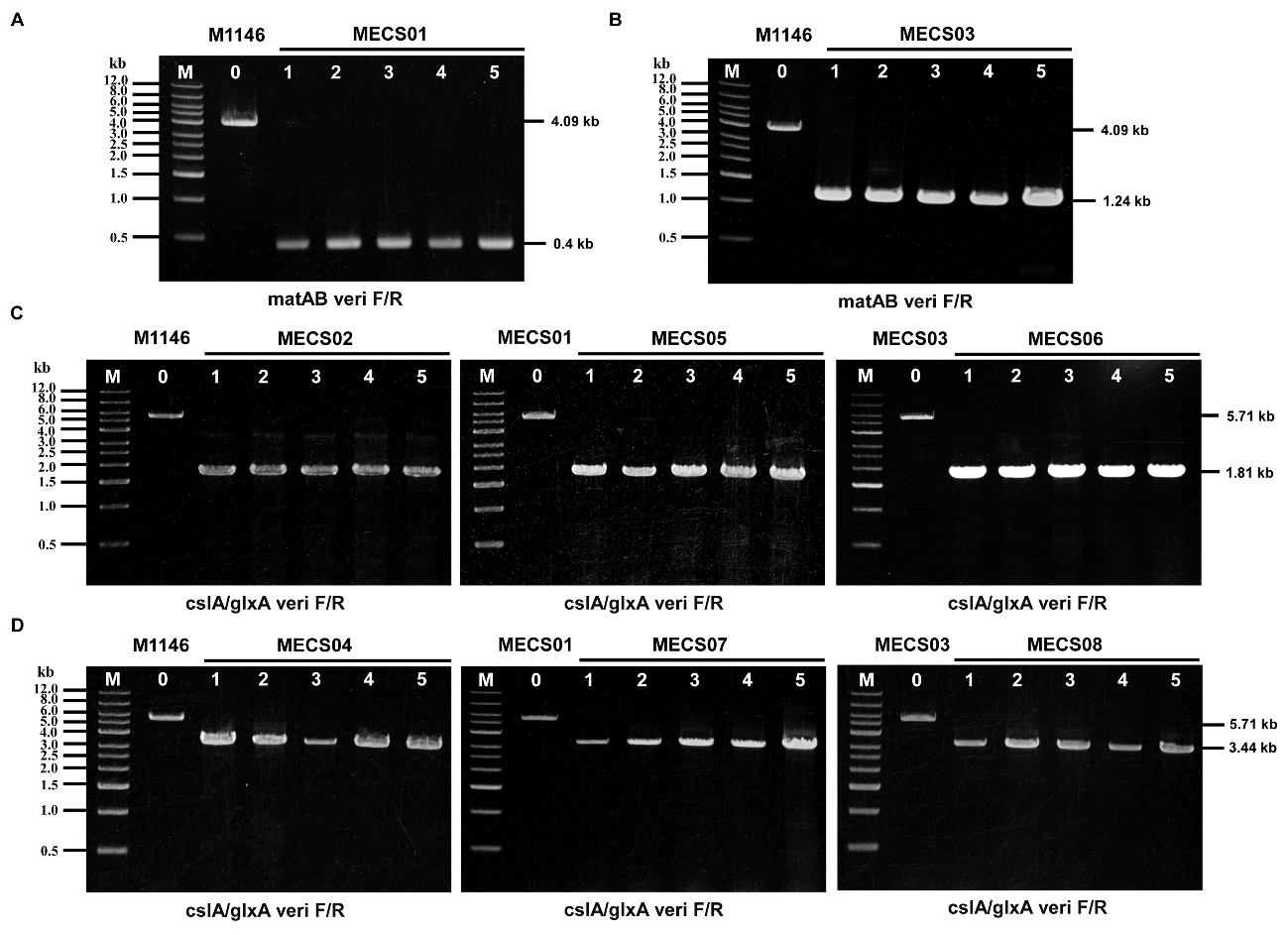
**

**Figure S2.** **Verification of the engineered strains by PCR amplifications.** The expected sizes of PCR amplicons are as indicated. “kb” represents kilobase, and “M” denotes DNA Ladder. Five transformants (1-5) of each engineered strain were randomly selected for DNA extractions and subsequent PCR amplifications. The parental strain (0) used in CRISPR/Cas9-mediated engineering was included to serve as a control.


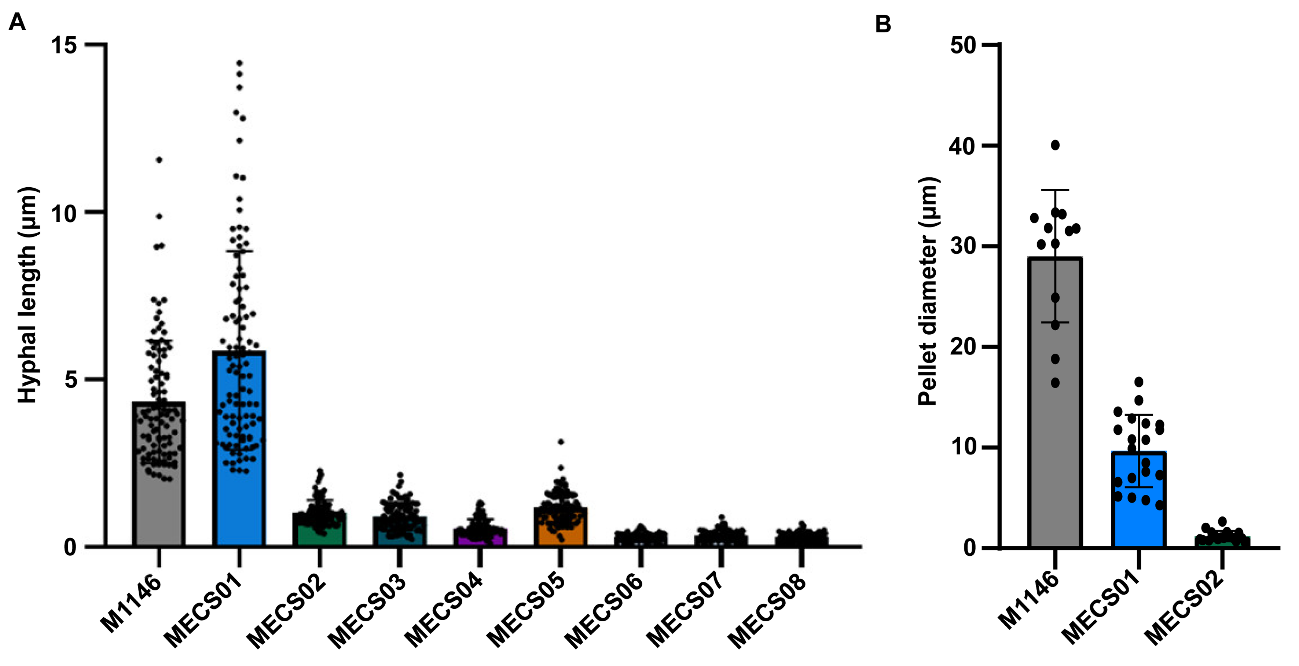


**Figure S3. Mycelial length and pellet diameter of *S. coelicolor* M1146 and its engineered derivatives.** (A) Mycelial length of *S. coelicolor* M1146 and the engineered strains MECS01~MECS08 after 3 days of culture in YEME liquid medium. For each strain, 100 hyphae were randomly selected and measured. (B) Pellet diameter of *S. coelicolor* M1146, MECS01 and MECS02 under the same culture conditions. All visible pellets in the field of view were measured: 13 pellets for *S. coelicolor* M1146, 20 for MECS01, and 17 for MECS02. No measurable pellets were observed for MECS03~MECS08. Scatter points indicate individual measurements, and error bars represent standard deviations.

**
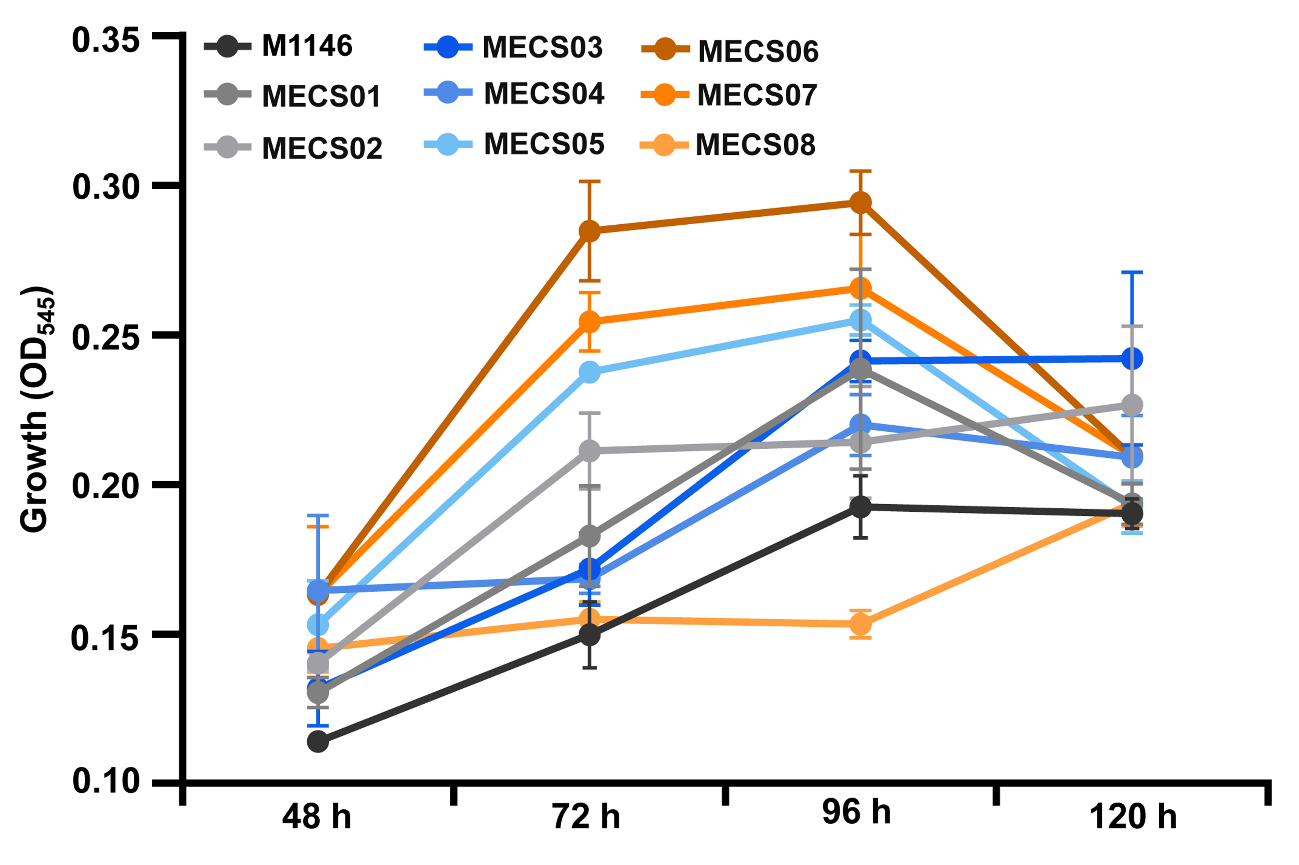
**

**Figure S4. Growth curves of *S. coelicolor* M1146 and its derivatives cultivated in YEME.** Spore suspensions of *S. coelicolor* M1146 and the engineered strains were used to inoculate 10 mL of liquid YEME for seed culture preparation. After incubation of 48 h, 1.0 mL of the seed culture was transferred into 50 mL of fresh YEME. Samples (0.5 mL each) were collected at various time points to measure cell growth. Data are presented as the averages of the results of three independent experiments. Error bars show standard deviations.

**
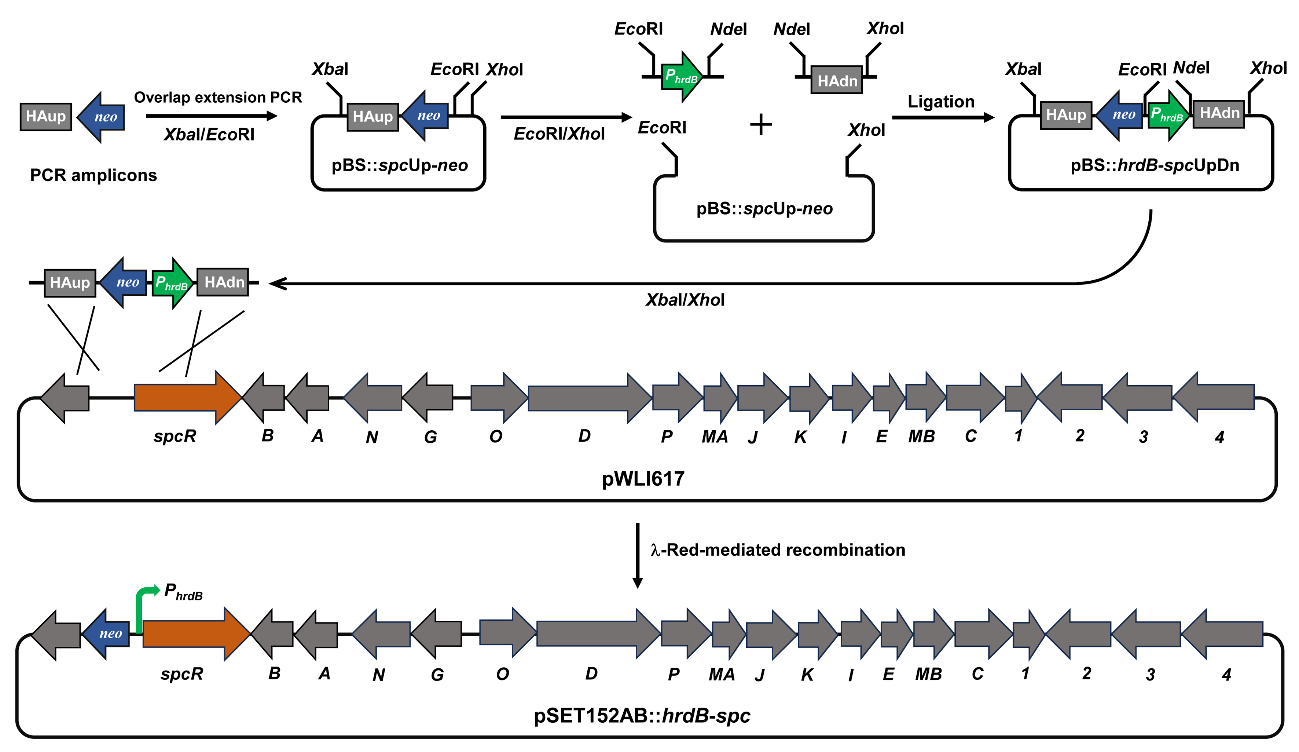
**

**Figure S5.** **A simplified schematic diagram for the construction of pSET152AB::*hrdB-spc*.** The procedure for constructing pBS::*hrdB*-*spc*UpDn was described in detail in the Materials and Methods. The fragment encompassing the upstream and downstream regions, the *neo* cassette and the *hrdB* promoter was released from pBS::*hrdB*-*spc*UpDn using *Xba*I/*Xho*I digestion. The fragment was then used to replace the native promoter of *spcR* in pWLI617 with the constitutive *hrdB* promoter *via* λ-Red-mediated recombination technology.

# Supplementary Tables

**Table S1.** Bacterial strains used in this study.

| **Bacteria strains** | **Relevant Characteristics** | **Reference/Source** |
| --- | --- | --- |
| ***Streptomyces* Strains** | | |
| *S. coelicolor* M1146 (abbreviated as M1146) | An engineered derivative of *S. coelicolor* M145 lacking gene clusters for actinorhodin (*act*), undecylprodigiosin (*red*), calcium dependent antibiotic (*cda*) and coelimycin P1 (*cpk*) | [1] |
| MECS01 | A mutant stain with the deletion of *matAB* in *S. coelicolor* M1146 | This work |
| MECS02 | A mutant stain with the deletion of *cslA/glxA* in *S. coelicolor* M1146 | This work |
| MECS03 | A mutant stain with the deletion of *matAB* and simultaneous overexpression of *ssgA* in *S. coelicolor* M1146 | This work |
| MECS04 | A mutant stain with the deletion of *cslA/glxA* and simultaneous overexpression of *ftsZ* in *S. coelicolor* M1146 | This work |
| MECS05 | A mutant stain with the deletion of both *matAB* and *cslA/glxA* in *S. coelicolor* M1146 | This work |
| MECS06 | A mutant stain with the deletion of both *matAB* and *cslA/glxA*, and simultaneous overexpression of *ssgA* in *S. coelicolor* M1146 | This work |
| MECS07 | A mutant stain with the deletion of both *matAB* and *cslA/glxA*, and simultaneous overexpression of *ftsZ* in *S. coelicolor* M1146 | This work |
| MECS08 | A mutant stain with the deletion of both *matAB* and *cslA/glxA*, and simultaneous overexpression of both *ssgA* and *ftsZ* in *S. coelicolor* M1146 | This work |
| M1146-*act* | A derivative of *S.* *coelicolor* M1146 containing pSET152-*act*, Apr^R^ | [2] |
| MECS01-*act* | A derivative of MECS01 containing pSET152-*act*, Apr^R^ | This work |
| MECS02-*act* | A derivative of MECS02 containing pSET152-*act*, Apr^R^ | This work |
| MECS03-*act* | A derivative of MECS03 containing pSET152-*act*, Apr^R^ | This work |
| MECS04-*act* | A derivative of MECS04 containing pSET152-*act*, Apr^R^ | This work |
| MECS05-*act* | A derivative of MECS05 containing pSET152-*act*, Apr^R^ | This work |
| MECS06-*act* | A derivative of MECS06 containing pSET152-*act*, Apr^R^ | This work |
| MECS07-*act* | A derivative of MECS07 containing pSET152-*act*, Apr^R^ | This work |
| MECS08-*act* | A derivative of MECS08 containing pSET152-*act*, Apr^R^ | This work |
| M1146-*spc* | A derivative of *S.* *coelicolor* M1146 containing pSET152AB::*hrdB*-*spc*, Apr^R^ | This work |
| MECS01-*spc* | A derivative of MECS01 containing pSET152AB::*hrdB*-*spc*, Apr^R^ | This work |
| MECS03-*spc* | A derivative of MECS03 containing pSET152AB::*hrdB*-*spc*, Apr^R^ | This work |
| MECS05-*spc* | A derivative of MECS05 containing pSET152AB::*hrdB*-*spc*, Apr^R^ | This work |
| M1146-*crt* | A derivative of *S.* *coelicolor* M1146 containing pSET152-*hrdB*-*crt*, Apr^R^ | This work |
| MECS01-*crt* | A derivative of MECS01 containing pSET152-*hrdB*-*crt*, Apr^R^ | This work |
| MECS03-*crt* | A derivative of MECS03 containing pSET152-*hrdB*-*crt*, Apr^R^ | This work |
| MECS05-*crt* | A derivative of MECS05 containing pSET152-*hrdB*-*crt*, Apr^R^ | This work |
| ***E. coli* Strains** |  |  |
| DH5α | F^–^ φ80*lac*ZΔM15 Δ(*lac*ZYA-*arg*F) U169 *rec*A1 *end*A1 *hsd*R17(r_K_^–^, m_K_^+^) *pho*A *sup*E44 λ^–^ *thi*-1 *gyr*A96 *rel*A1 | ThermoFisher Scientific |
| ET12567(pUZ8002) | *dam dcm hsdS cat tet/*pUZ8002 | [3] |
| BW25113 | K-12 derivative; ΔaraBAD ΔrhaBAD | [4] |

Apr^R^, apramycin resistance.

**Table S2.** Plasmids used in this study.

| **Plasmids** | **Relevant Characteristics** | **Reference/Source** |
| --- | --- | --- |
| pKCcas9dO | *acc(3)IV*, pSG5, *tipA*-*Scocas9*, Apr^R^ | [5] |
| pKCcas9::Δ*matAB* | A derivative of pKC1139 containing *Cas9* driven by the *tipA* promoter, sgRNA targeting *matA* and homologous regions flanking *matAB*, Apr^R^ | This work |
| pKCcas9::Δ*cslA*/*glxA* | A derivative of pKC1139 containing *Cas9* driven by the *tipA* promoter, sgRNA targeting *cslA* and homologous regions flanking *cslA/glxA*, Apr^R^ | This work |
| pKCcas9::Δ*matAB*-*ssgA*OE | A derivative of pKC1139 containing *Cas9* driven by the *tipA* promoter, sgRNA targeting *matA*, homologous regions flanking *matAB*, and *ssgA* under the control of the *hrdB* promoter, Apr^R^ | This work |
| pKCcas9::Δ*cslA*/*glxA*-*ftsZ*OE | A derivative of pKC1139 containing *Cas9* driven by the *tipA* promoter, sgRNA targeting *cslA*, homologous regions flanking *cslA/glxA*, and *ftsZ* under the control of the *hrdB* promoter, Apr^R^ | This work |
| pBluescript II KS (+) | Routine cloning and subcloning vector, Amp^R^ | Stratagene |
| pSET152 | Integrative vector, Apr^R^ | [6] |
| pSET152::*act* | A derivative of pSET152 containing the entire *act* gene cluster, Apr^R^ | [2] |
| pWLI617 | A modified cosmid harboring the entire *spc* gene cluster from *S. sanyensis* FMA, *φC31 attP/int*, *oriT*, Integrative vector, Apr^R^ | [7] |
| pBS::*hrdB-spc*UpDn | A derivative of pBluescript II KS (+) containing homologous region *spc* gene cluster driven by the *hrdB* promoter, Amp^R^ | This work |
| pSET152AB::*hrdB*-*spc* | A derivative of pWLI617 containing an engineered *spc* gene cluster with *spcR* driven by the *hrdB* promoter, Apr^R^ | This work |
| pBS::*crt* | A derivative of pBluescript II KS (+) containing the entire *crt* gene cluster, Amp^R^ | This work |
| pSET152::*hrdB-crt*UpDn | A derivative of pSET152 containing upstream and downstream regions of the *crt* gene cluster used for λ-mediated recombination, Apr^R^ | This work |
| pSET152::*hrdB*-*crt* | A derivative of pSET152 containing an engineered *crt* gene cluster with the promoters of *crt*E and *crt*Y replaced by the constitutive *hrdB* promoter, Apr^R^ | This work |
| Apr^R^, apramycin resistance; Amp^R^, ampicillin resistance. | | |

**Table S3.** Primers used in this study.

| **Primers** | **Sequence (5′-3′)**^a,b^ | **Purpose** |
| --- | --- | --- |
| matAB UpF | *GAGTCGGTGCTTTTTTTGAG*GATCATCACGACGGCGCTGAC | Construction of pKCcas9::Δ*matAB* |
| matAB UpR | aattCTTAAGCGAGCGCCCCCTGTATCACAC | Construction of pKCcas9::Δ*matAB* |
| matAB DnF | aattCTTAAGGAGGCCGGTCGGATGACCAC | Construction of pKCcas9::Δ*matAB* |
| matAB DnR | aattAAGCTTCTTCACCGAGGACATCCGGCTG | Construction of pKCcas9::Δ*matAB* |
| matAB sgRNA F | aattACTAGTCTCGGAGTCGTATGCGCACCGTTTTAGAGCTAGAAATAG | Construction of pKCcas9::Δ*matAB* |
| sgRNA R | CTCAAAAAAAGCACCGACTC | Construction of pKCcas9::Δ*matAB* and pKCcas9::Δ*cslA*/*glxA*-*ftsZ*OE |
| matAB hrdB pF | aattTCTAGA*TGTGATACAGGGGGCGCTCG*CCGCCTTCCGCCGGAACGG | Construction of pKCcas9::Δ*matAB*-*ssgA*OE |
| hrdB pR | aattCATATGGAACAACCTCTCGGAACGTTGAAAAACGGC | Construction of pKCcas9::Δ*matAB*-*ssgA*OE and pKCcas9::Δ*cslA*/*glxA*-*ftsZ*OE |
| ssgA F | aattCATATGATGAGCTTTCTCGTGTCCGAGG | Construction of pKCcas9::Δ*matAB*-*ssgA*OE |
| ssgA R | aattGAATTC*GTGGTCATCCGACCGGCCTC*TCAGCCCGCGCTCTGTTCCT | Construction of pKCcas9::Δ*matAB*-*ssgA*OE |
| matAB veri F | GTGAGGGATGGATTCCGGCC | Verification of mutants |
| matAB veri R | GAAGGGCGTGGTCGGAAAAG | Verification of mutants |
| cslA/glxA UpF | *GAGTCGGTGCTTTTTTTGAG*ACCCGGCCCCCTCGTGTAGTAC | Construction of pKCcas9::Δ*cslA*/*glxA* |
| cslA/glxA UpR | aattCTTAAGGTCGGCGTCGACGTCATGAGT | Construction of pKCcas9::ΔcslA/glxA |
| cslA/glxA DnF | aattCTTAAGCGCGGGTTGTGTTTCGGGTGC | Construction of pKCcas9::Δ*cslA*/*glxA* |
| cslA/glxA DnR | aattAAGCTTGAGCGTGCCGTCTGCTGGGAC | Construction of pKCcas9::Δ*cslA*/*glxA* |
| cslA/glxA sgRNA F | aattACTAGTGTCCTGGTTGTTGCCGGACCGTTTTAGAGCTAGAAATAG | Construction of  pKCcas9::Δ*cslA*/*glxA* |
| cslA/glxA hrdB pF | aattTCTAGA*CTCATGACGTCGACGCCGAC*CCGCCTTCCGCCGGAACGG | Construction of  pKCcas9::Δ*cslA*/*glxA*-*ftsZ*OE |
| ftsZ F | ttaaCATATGTGGCAGCACCGCAGAACTAC | Construction of  pKCcas9::Δ*cslA*/*glxA*-*ftsZ*OE |
| ftsZ R | aattGAATTC*CACCCGAAACACAACCCGCG*TCACTTCAGGAAGTCCGGCA | Construction of  pKCcas9::Δ*cslA*/*glxA*-*ftsZ*OE |
| cslA/glxA veri F | CGTAGAACCCTGGTCCGGTTGG | Verification of mutants |
| cslA/glxA veri R | CCGCACCACGTGCACCGATC | Verification of mutants |
| spc UpF | ttaaTCTAGAGCGCCCGGTCAGTACGCCTC | Construction of pBS::*hrdB-spc*UpDn |
| spc UpR | GTGAACGTCGAGGCGACGGC | Construction of pBS::*hrdB-spc*UpDn |
| spc DnF | aattCATATGATGGGTCCTCAGGTACGAGC | Construction of pBS::*hrdB-spc*UpDn |
| spc DnR | aattCTCGAGAGGTCGTCGAGCGCCACGAC | Construction of pBS::*hrdB-spc*UpDn |
| spc neo F | *GCCGTCGCCTCGACGTTCAC*ACTAGTTCAGAAGAACTCGTCAAGAA | Construction of pBS::*hrdB-spc*UpDn |
| spc neo R | ttaaGAATTCACTAGTCCTGGATACCGCTCGCCGCA | Construction of pBS::*hrdB-spc*UpDn |
| spc hrdB pF | aattGAATTCCCGCCTTCCGCCGGAACGGC | Construction of pBS::*hrdB-spc*UpDn |
| spc hrdB pR | ttaaCATATGGAACAACCTCTCGGAACGTTGAAAAACGGC | Construction of pBS::*hrdB-spc*UpDn |
| crt 1F | ttaaTCTAGAGTGACACCCCGTTCCGCCCAGG | Construction of pBS::*crt* |
| crt 1R | aattAAGCTTGCGGGGTGAGGCGGTCCAGGC | Construction of pBS::*crt* |
| crt 2F | ttaaTCTAGAAGTACTACAACCTCCGCGGACTGCTC | Construction of pBS::*crt* |
| crt 2R | aattAAGCTTGAACGGGCCGGGAACCGGTGG | Construction of pBS::*crt* |
| crt 3F | ttaaTCTAGACACGTGGTCGATGTCGTACCGG | Construction of pBS::*crt* |
| crt 3R | aattAAGCTTGTGCGGACAACCGAGAGTGAGA | Construction of pBS::*crt* |
| crt UpF | *GCCGTTTTTCAACGTTCCGAGAGGTTGTTC*GTGACACCCCGTTCCGCCCAG | Construction of pSET152::*hrdB-crt*UpDn |
| crt UpR | ttaaAGATCTCAGCAGACACGTACGCGCCGG | Construction of pSET152::*hrdB-crt*UpDn |
| crt DnF | *GCCGTTTTTCAACGTTCCGAGAGGTTGTTC*GTGCGGACAACCGAGAGTGAG | Construction of pSET152::*hrdB-crt*UpDn |
| crt DnR | aattAGATCTCGTCTCGGTCAGCAGGTCGTC | Construction of pSET152::*hrdB-crt*UpDn |
| hrdB UpF | ttaaTCTAGACCGCCTTCCGCCGGAACGGC | Construction of pSET152::*hrdB-crt*UpDn |
| hrdB DnF | aattGAATTCCCGCCTTCCGCCGGAACGGC | Construction of pSET152::*hrdB-crt*UpDn |
| hrdB crt pR | GAACAACCTCTCGGAACGTTGAAAAACGGC | Construction of pSET152::*hrdB-crt*UpDn |

^a^ Underlined sequences for restriction enzyme recognition sites; ^b^ italicized sequences for overlapping between DNA sequences.

**Supplementary References**

[1] Gomez-Escribano JP, Bibb MJ. Engineering *Streptomyces* *coelicolor* for heterologous expression of secondary metabolite gene clusters. Microb Biotechnol 2011;4:207-15. <https://doi.org/10.1111/j.1751-7915.2010.00219.x>

[2] Wang X, Fu Y, Wang M, Niu G. Synthetic cellobiose-inducible regulatory systems allow tight and dynamic controls of gene expression in *Streptomyces*. ACS Synth Biol 2021;10:1956-65. <https://doi.org/10.1021/acssynbio.1c00152>

[3] Paget MS, Chamberlin L, Atrih A, Foster SJ, Buttner MJ. Evidence that the extracytoplasmic function sigma factor σ^E^ is required for normal cell wall structure in *Streptomyces coelicolor* A3(2). J Bacteriol 1999;181:204-11. <https://doi.org/10.1128/jb.181.1.204-211.1999>

[4] Datsenko KA, Wanner BL. One-step inactivation of chromosomal genes in *Escherichia coli* K-12 using PCR products. Proc Natl Acad Sci U S A 2000;97:6640-5. <https://doi.org/10.1073/pnas.120163297>

[5] Huang H, Zheng G, Jiang W, Hu H, Lu Y. One-step high-efficiency CRISPR/Cas9-mediated genome editing in *Streptomyces*. Acta Biochim Biophys Sin 2015;47:231-43. <https://doi.org/10.1093/abbs/gmv007>

[6] Bierman M, Logan R, O'Brien K, Seno ET, Rao RN, Schoner BE. Plasmid cloning vectors for the conjugal transfer of DNA from *Escherichia coli* to *Streptomyces* spp. Gene 1992;116:43-9. <https://doi.org/10.1016/0378-1119(92)90627-2>

[7] Li T, Du Y, Cui Q, Zhang J, Zhu W, Hong K, *et al*. Cloning, characterization and heterologous expression of the indolocarbazole biosynthetic gene cluster from marine-derived *Streptomyces sanyensis* FMA. Mar Drugs 2013;11:466-88. <https://doi.org/10.3390/md11020466>
